# Transfer partizipativer Forschungsergebnisse in die Praxis: Das Beratungsinstrument *Standortanalyse* in der kommunalen Gesundheitsförderung

**DOI:** 10.1007/s00103-020-03273-x

**Published:** 2021-01-05

**Authors:** Petra Wihofszky, Petra Hofrichter, Sandra Layh, Mareen Jahnke

**Affiliations:** 1 Institut für Gesundheits- und Pflegewissenschaften, Fakultät Soziale Arbeit, Gesundheit und Pflege, Hochschule Esslingen, Flandernstraße 101, 73732 Esslingen a. Neckar, Deutschland; 2 Hamburgische Arbeitsgemeinschaft für Gesundheitsförderung e. V., 20097 Hamburg, Deutschland; 3 Fakultät Soziale Arbeit, Gesundheit und Pflege, Hochschule Esslingen, 73732 Esslingen a. Neckar, Deutschland; 4 vdek-Landesvertretung Hamburg, 20097 Hamburg, Deutschland

**Keywords:** Forschungsgemeinschaft, Sozialraum, Fachkraft, Evaluation, Vernetzung, Research community, Community, Professional, Evaluation, Networking

## Abstract

In Hamburg wird der Auf- und Ausbau von integrierten kommunalen Gesundheitsstrategien in Sozialräumen gefördert. Für die Beratung entwickelten wir, die Forschungsgemeinschaft des Projekts „Kommunale Entwicklung von Gesundheitsstrategien“, in einem partizipativen Prozess das Instrument „Standortanalyse“. Die Konzeption gründet auf Ergebnissen des Projekts und orientiert sich am Modell „Community Readiness“. Die Standortanalyse dient der Einschätzung eines Sozialraums und richtet sich an Fachkräfte. Dieser Beitrag beschreibt, wie ein Transfer in die Praxis gelingen kann. Hierzu werden Ergebnisse der Erprobungsphase der Standortanalyse, die wir von 2019 bis 2020 in 4 Stadtteilen Hamburgs evaluativ begleiteten, vorgestellt.

Die Standortanalyse umfasst ein Tableau, Themenfelder und Fragekarten sowie ein Arbeitsheft. Im Mittelpunkt steht die Analyse der Themenfelder. Gegenstand unserer qualitativen Evaluation waren die Akzeptanz der Beratungen, die Passung und Handhabung des Instruments sowie mögliche Weiterentwicklungsbedarfe.

Die Ergebnisse gliedern sich in Beratungsvorgehen, Beratungskontext und Wirkungen. Zentrale Erkenntnisse sind: Das Instrument fördert die Vernetzung von Fachkräften, trägt zur Strukturierung und Transparenz in der Entwicklung von integrierten kommunalen Strategien in Sozialräumen bei, schließt Wissenslücken und unterstützt das Handling der Prozesse. Für die Praxis ist eine externe Prozessbegleitung bedeutsam. Neben der Verfügbarkeit eines Instruments ist es künftig wichtig, in die Qualifikation von Beratenden sowie in digitale Angebote zu investieren.

## Abstract (en)

Hamburg promotes the establishment and expansion of integrated municipal strategies in communities. For the consultation, we (the research community of the “Community Development of Health Strategies” project) developed a tool called *Standortanalyse* (*R*eadiness for Establishing and Expanding *I*ntegrated *M*unicipal *S*trategies of Health Promotion [RIMS]) using a participatory process. The tool design is based on the project’s findings and follows the community readiness model*.* *Standortanalyse* is a location analysis that helps assess a community and is aimed at professionals. This article describes how translation into practice can be achieved. To that end, we present the findings of the *Standortanalyse* pilot phase in four districts of Hamburg from 2019 to 2020, which we accompanied with an evaluation.

The *Standortanalyse* tool consists of a board, topic areas, question cards, and a workbook. The tool’s central element is the analysis of the topic areas. The subject of our qualitative evaluation was acceptance of the consultations, suitability and handling of the tool itself, and needs for further development.

The results have been broken down to consultation context, consultation approach, and effects. The central findings are: the tool promotes networking among professionals, contributes to structuring and transparency in the development of integrated municipal strategies in communities, closes knowledge gaps, and supports the processes. In practice, external process support is significant. In addition to the availability of the tool, it will be important to invest in consultant training and digital services in the future.

## Hintergrund

Gesundheitsförderung setzt in Kommunen sozialraumorientiert an [1–3]. Mit Kommune ist nicht lediglich eine Verwaltungseinheit gemeint, sondern der Begriff steht für soziale Räume, in denen gesundheitsfördernde Maßnahmen stattfinden [4]. Die Kommune nimmt dabei die Funktion ein, Akteure wie z. B. Kitas und Schulen miteinander zu verbinden. Die Idee, akteursübergreifend kommunale Strategien aufzubauen, entstand in der Kinder- und Jugendhilfe [5, 6]. Inzwischen beziehen sich sogenannte integrierte kommunale Strategien (IKS) in der Gesundheitsförderung auf die gesamte Lebensspanne, von der Kindheit und Jugend bis ins hohe Erwachsenenalter [4–6]. IKS werden auch als Präventionsketten bezeichnet [6], deren Ziel darin besteht, „dass alle Angebote, Maßnahmen und Träger zusammengeführt werden und alle Akteure vor Ort entlang einer gemeinsamen Strategie für die Gesundheit der Bewohner in allen Lebensphasen zusammenarbeiten“ [4]. An der Entwicklung einer IKS sind Fachkräfte unterschiedlicher Ressorts und Aufgabenbereiche, einschließlich freier Träger, sowie auch Bürger*innen aus Selbsthilfe- und Stadtteilinitiativen und Netzwerken beteiligt. Eine wesentliche Herausforderung besteht darin, Fachkräfte in ihren unterschiedlichen beruflichen Rollen, Zuständigkeiten und Aufgabenfeldern zusammenzubringen. Der gemeinsame Bezugspunkt beim Aufbau einer IKS sind die Bewohnerschaft und das Bestreben, an gesundheitlichen Einflussfaktoren anzusetzen und Bedingungen zu schaffen, die das Wohlbefinden und die Lebensqualität der Menschen in ihrem Wohnumfeld fördern.

Der Auf- und Ausbau von IKS wird in der kommunalen Gesundheitsförderung noch als eine „junge Entwicklung“ bewertet [4] – auch wenn Praxisbeispiele beschrieben wurden sowie Handreichungen und Arbeitshilfen entstanden sind. Die Landesvereinigung für Gesundheit und Akademie für Sozialmedizin Niedersachsen e. V. (LVG & AFS) entwickelte das Programm Präventionsketten Niedersachsen sowie das Projekt „Kontextcheck“ [7] und bietet z. B. Kommunen Hilfestellungen zum Ablauf und verschiedene Ausgestaltungsmöglichkeiten von kommunaler Gesundheitsförderung. Die Handreichung des Deutschen Instituts für Urbanistik liefert Erkenntnisse zu Rahmenbedingungen und zur Steuerung von IKS sowie Hinweise zu erfolgreicher Kooperation [8]. Auf den Seiten des Kooperationsverbunds „Gesundheitliche Chancengleichheit“ finden sich Veröffentlichungen, die die Grundlagen zur Verbesserung gesundheitlicher Chancengleichheit thematisieren, so auch zum Thema kommunale Strategien. Die Handreichung „Auf dem Weg zu gesunden Landkreisen und Städten“ beschreibt Gelingensfaktoren, Stolpersteine und mögliche passende Lösungen für die Gestaltung von IKS entlang der 3 Phasen „Auftauen“, „Umsetzen“ und „Verstetigen“ [9]. Ein Ergebnisbericht des GKV-Bündnisses zur Evidenzlage von IKS gibt Handlungsempfehlungen auf Basis eines durchgeführten Reviews [10]. Mit der Umsetzung des Präventionsgesetzes haben die gesetzlichen Krankenkassen und die Länder ihre Aktivitäten zum Ausbau von gesundheitsfördernden Strukturen und Maßnahmen sowie auch von IKS gestärkt. Insbesondere stellt das kommunale Förderprogramm des GKV-Bündnisses Mittel für den Strukturaufbau zur Verfügung [4]. Deshalb sollte aus unserer Sicht der Erforschung von IKS zukünftig mehr Rechnung getragen werden.

Das Forschungsprojekt „Kommunale Entwicklung von Gesundheitsstrategien“ (KEG) untersuchte mit partizipativen Methoden den Auf- und Ausbau von IKS im Hamburger Stadtteil Rothenburgsort. KEG ist Teil des Forschungsverbundes PartKommPlus, der wiederum Teil der partizipativen Gesundheitsforschung (PGF) ist [11]. Unter PGF wird ein Prozess verstanden, bei dem Beteiligte aus Wissenschaft, Praxis und Zivilgesellschaft miteinander forschen [12]. In sogenannten Forschungsgemeinschaften sollen Erkenntnisse gewonnen werden, die zur Förderung von Gesundheit und Wohlbefinden derjenigen beitragen, deren Leben und Arbeit im Mittelpunkt der Forschung stehen. PGF unterscheidet sich von konventioneller Forschung in dieser doppelten Zielsetzung: Ziel ist es, nicht nur Erkenntnisse zu gewinnen, sondern auch Impulse für Veränderungen in der Praxis zu setzen [13]. Im Werteverständnis von PGF stehen insbesondere die Lebensbedingungen sozial benachteiligter Gruppen im Mittelpunkt [14]. Die Forschungsgemeinschaft KEG, die die Entwicklung einer IKS in Rothenburgsort erforschte, setzte sich aus Mitarbeitenden der Hamburgischen Arbeitsgemeinschaft für Gesundheitsförderung e. V. (HAG), der Behörde für Gesundheit und Verbraucherschutz (seit Juli 2020 Behörde für Arbeit, Gesundheit, Soziales, Familie und Integration, kurz: Sozialbehörde), des Bezirks Hamburg-Mitte und der Hochschule Esslingen zusammen.

Der Aufbau einer IKS begann in Rothenburgsort mit der Gründung eines Netzwerks von Fachkräften, das sich zum Ziel gesetzt hatte, Entwicklungsbedingungen von Kindern und deren Familien zu fördern. Rothenburgsort gehört zum Bezirk Hamburg-Mitte und zählt zu den ärmsten Stadtteilen Hamburgs [15, 16]. Der Stadtteil wurde als Modellstandort für eine IKS im Rahmen der landesweiten Präventionsstrategie „Pakt für Prävention“ ausgewählt [17]. Nach einer erfolgreichen Auftaktphase ließ in den darauffolgenden Jahren die Bereitschaft der Fachkräfte nach, sich einzubringen. Im Sinne der doppelten Zielsetzung von PGF stellten wir uns als Forschungsgemeinschaft einerseits die Frage, welche Bedingungen die Entwicklung einer IKS beeinflussen, und zum anderen auch, wie der Auf- und Ausbau der IKS in Rothenburgsort redynamisiert werden könnte. Dazu befragten wir im Jahr 2016 aktiv beteiligte sowie auch aus dem Netzwerk ausgeschiedene Fachkräfte und Bewohner*innen aus Rothenburgsort. Wir arbeiteten mit der Methode Appreciative Inquiry (übersetzt: wertschätzende Erkundung; [18–20]): Wir fragten nicht nach Defiziten und Fehlern, sondern danach, was positiv in der Netzwerkarbeit erlebt worden war und welche Erwartungen die Beteiligten an diese Arbeit gehabt hatten [21]. Unsere Ergebnisse zeigten, dass Entscheidungsträger*innen und Fachkräfte dafür sensibilisiert werden müssen, wie sie selbst den Auf- und Ausbau von IKS befördern können. 2 Ansatzpunkte sind wesentlich: Erstens müssen die Nutzenden einer IKS eine Stimme erhalten und gehört werden; ihre Vorschläge müssen möglichst zeitnah umgesetzt werden. Zweitens braucht es Qualifizierungsangebote für die beteiligten Fachkräfte und die Möglichkeit, den Prozess durch Beratung und Coaching zu begleiten. Um an beiden Punkten ansetzen zu können, entwickelten wir auf der Grundlage unserer Befragungsergebnisse das Beratungsinstrument „Standortanalyse für den Auf- und Ausbau integrierter kommunaler Strategien“ von 2018 bis 2019. Mit der Standortanalyse sollte der Koordinierungsstelle Gesundheitliche Chancengleichheit (KGC) in Hamburg ein Instrument an die Hand gegeben werden, um die Situation in einem Sozialraum gemeinsam mit Fachkräften zu reflektieren und einzuschätzen. Der Transfer partizipativer Forschungsergebnisse in die Praxis mittels eines Beratungsinstrumentes ist Gegenstand dieses Beitrags.

## Das Beratungsinstrument „Standortanalyse“

Die Standortanalyse ist ein Instrument zur Begleitung und Beratung von Fachkräften, die eine IKS aufbauen oder weiterentwickeln (wollen). Nach der Entwicklung holten wir als Pretest ein kollegiales Feedback ein. Anschließend wurde die Standortanalyse von Januar 2019 bis Januar 2020 bei der Beratung von 4 Hamburger Stadtteilen eingesetzt und wurde von uns als Forschungsgemeinschaft evaluativ begleitet. Im Folgenden beschreiben wir das Instrument Standortanalyse und erläutern das methodische Vorgehen in der Evaluation.

**Figure 1:**
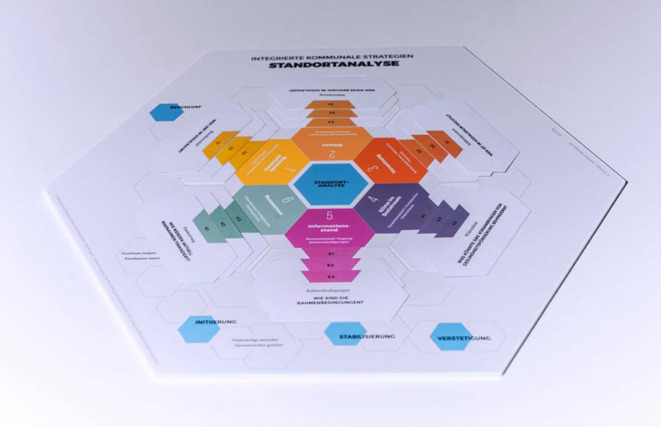

Die Standortanalyse setzt sich aus folgenden Bestandteilen zusammen: dem Tableau, den Themenfeldern mit den zugehörigen Fragekarten sowie einem Arbeitsheft. Das Tableau hat die Form einer Wabe, kann in der Mitte des Tisches platziert werden und ist drehbar (Abb. 1). In der Mitte dieser Wabe steht der Begriff „Standortanalyse“ – der Teil der Vorbereitung ist, bevor die Planung einer IKS erfolgt. Die Phasen davor und danach werden auf dem Tableau als kleinere Waben dargestellt: Vor dem Start der Arbeit mit der Standortanalyse ist ein Beschluss für eine IKS auf kommunaler Ebene aus unserer Sicht empfehlenswert, aber nicht notwendig. Die Phasen Initiierung, Stabilisierung und Verstetigung schließen sich nach der Durchführung der Standortanalyse an.

Den Schwerpunkt der Standortanalyse bilden 6 Themenfelder. Sie leiten sich aus den Ergebnissen der Befragung in Rothenburgsort ab. Nach unseren Befragungsergebnissen sind förderliche Bedingungen für den Aufbau einer IKS [21]:

- die soziale und gesundheitliche Lage der Bewohner*innenschaft zu kennen,
- sich Ziele zu setzen,
- den Sozialraum überschaubar zu halten,
- sich einen Überblick über die Netzwerkstrukturen zu verschaffen,
- Möglichkeiten für eine Koordinationsstelle zu suchen,
- sich mit den Anforderungen an Koordination auseinanderzusetzen,
- die Verfügbarkeit von Ressourcen zu prüfen und
- sich ein Bild zu machen, wer vor Ort ist und sich einbringen will.

Diese Bedingungen übersetzten wir in die 6 Themenfelder: Ausgangssituation, Wissen, Vernetzung, Klima im Sozialraum, Ressourcen und Informationsstand. Die Themenfelder stellen die zentralen Kriterien für die Einschätzung eines Sozialraums dar und werden in Tab. 1 detailliert aufgeführt und erläutert.

**Table 1:** 

1	Ausgangssituation	Die Merkmale der im Sozialraum lebenden Bewohnerschaft und die bestehenden gesundheitsfördernden Angebote werden unter Berücksichtigung der bestehenden Bedarfe erfasst
2	Wissen	Die Kenntnisse über bereits vorhandene gesundheitsfördernde Angebote im Sozialraum werden zusammengetragen und ihre Stärken und Schwächen abgewogen
3	Vernetzung	Vernetzung umfasst die Schlüsselakteur*innen und Netzwerke im Sozialraum, die für Gesundheitsförderung wichtig sind und sich dafür engagieren (könnten)
4	Klima im Sozialraum	Die Struktur der Zusammenarbeit der Fachkräfte und die Beteiligung der Bewohnerschaft an der Gesundheitsförderung werden thematisiert
5	Ressourcen	Die aktuellen Finanzierungsmöglichkeiten für gesundheitsfördernde Angebote werden benannt und zusätzlich verfügbare oder geplante materielle, personelle und sonstige Mittel festgehalten
6	Informationsstand	Wie viel und was die Fachkräfte im Sozialraum über das Thema IKS wissen, wird erörtert

**Figure 2:**
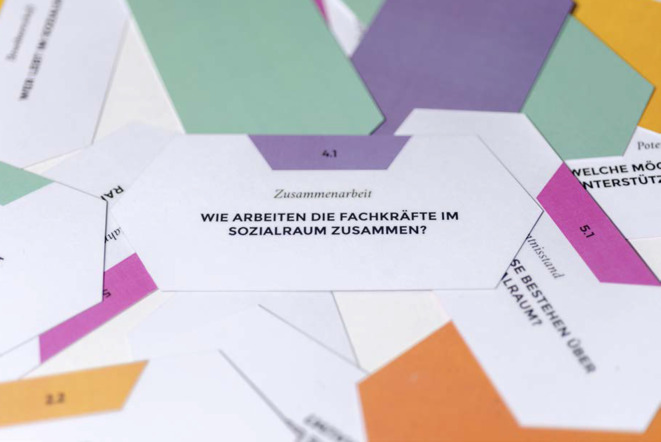

Die Themenfelder orientieren sich am theoretischen Modell Community Readiness [22]. Das Modell Community Readiness beschreibt Phasen, die durchlaufen werden, wenn Neuerungen in Stadtteilen eingeführt werden. Erst wenn eine gewisse „Handlungsbereitschaft“ oder „soziale Reife“ erreicht ist, lohnt es sich, nächste Schritte der Planung und Umsetzung anzugehen [23, 24]. Das Modell beschreibt verschiedene Dimensionen, die die Einschätzung eines Sozialraums erlauben. Entsprechend unseren Forschungsergebnissen aus Rothenburgsort haben wir davon ausgehend einen Rahmen für unser Instrument entwickelt. Während mit dem Modell Community Readiness mit einem strukturierten Leitfadeninterview die Situation eines Stadtteils per Momentaufnahme erfasst wird [22, 23], ist es das Ziel und Anliegen der Standortanalyse, Fachkräfte über einen Zeitraum zu begleiten und mithilfe der Dimensionen, die wir als Themenfelder bezeichnen, die Situation eines Sozialraums gemeinsam zu bewerten. Die Themenfelder in der Standortanalyse sind in ihrer Bedeutung gleichrangig. Jedes Themenfeld setzt sich wiederum aus 3 inhaltlichen Schwerpunkten zusammen, die auf insgesamt 18 Fragekarten als Gesprächsimpulse formuliert sind (Abb. 2). Auf der Grundlage der Analyse und Reflexion aller Fragekarten wird eine Einschätzung des Sozialraums vorgenommen. Anregungen zur Weiterarbeit ergänzen die Selbsteinschätzung.

Ein Arbeitsheft erläutert das Instrument Standortanalyse – seine Entstehung, Bestandteile sowie den Ablauf eines Beratungsprozesses. Darüber hinaus enthält das Arbeitsheft grafische Abbildungen, die einen Überblick über das Instrument geben sowie die Themenfelder und Fragekarten im Detail zeigen. Ein Beratungsleitfaden wurde als ein Ergebnis der in diesem Beitrag dargestellten Evaluation mit in das Arbeitsheft aufgenommen. Derzeit erarbeiten die Kooperationspartnerinnen an digitalen Formaten, die auf der Homepage der HAG angeboten werden. Das Instrument soll zukünftig auch bei Beratungsprozessen, die als Videokonferenzen durchgeführt werden, eingesetzt werden. Die digitale Weiterentwicklung erweitert die Vielfalt der Nutzung, unter Berücksichtigung der Bedarfe der Nutzer*innen bzw. der örtlichen Rahmenbedingungen (z. B. Abstandsgebote während der COVID-19-Pandemie).

### Evaluation

Im Mittelpunkt der Evaluation der Standortanalyse stand der Beratungsprozess. Evaluationsgegenstand waren die Akzeptanz der Beratungen, die Passung und Handhabung des Instruments sowie mögliche Weiterentwicklungsbedarfe des Angebots. Um Teilnehmende zu gewinnen, nutzten wir Kanäle wie Fachveranstaltungen, Kongresse, Arbeitsgruppen, Online- und Printmedien.

Zum Verständnis des Evaluationsgegenstandes werden die Schritte des Beratungsprozesses zum Zeitpunkt der Evaluation dargestellt: 1. Ein telefonisches oder persönliches Informationsgespräch führte die zuständige Mitarbeiterin der KGC in ihrer Rolle als Beraterin mit der/dem Koordinierenden eines interessierten Sozialraums. 2. Bei Interesse wurde ein erster Termin vereinbart. 3. Der Beratungsprozess, der in Gruppen mit ca. 5–8 Teilnehmenden stattfand, erstreckte sich je nach Bedarf über 2–3 Termine. Das Arbeitsheft wurde den Teilnehmenden beim ersten Termin ausgehändigt.

Während der Beratungen arbeiteten wir mit teilnehmenden Beobachtungen [25]. Hierzu begleitete die wissenschaftliche Mitarbeiterin des Projekts die Beraterin der KGC zu den einzelnen Beratungsterminen und hielt die Beobachtungen zum Umgang mit dem Instrument in einer Dokumentationsvorlage fest. Insgesamt erfolgten 11 teilnehmende Beobachtungen. 4 Gruppendiskussionen mit den Teilnehmenden führten wir nach Abschluss der Beratungen mit den Teilnehmenden durch [26]. Zudem wurden 4 Leitfadeninterviews mit der Beraterin (KGC) zu den einzelnen Beratungsprozessen durchgeführt [27].

Vor Beginn informierten wir die Teilnehmenden über die Evaluationsforschung und baten um Mitwirkung. Wir holten ein schriftliches Einverständnis ein. Die gewonnenen Daten wurden aufbereitet, transkribiert und in der Forschungsgemeinschaft mit Methoden der qualitativen Inhaltsanalyse ausgewertet und interpretiert [28]. Die Auswertung erfolgte computergestützt [29]. Im Rahmen dieses Auswertungsprozesses spiegelten und triangulierten wir die Erkenntnisse aus den verschiedenen Datenquellen und verdichteten sie für die Ergebnisdarstellung [27].

## Ergebnisse

Die Ergebnisse der Evaluation werden im Folgenden dargestellt und gliedern sich in die 3 Auswertungskategorien Beratungsvorgehen, Beratungskontext und Wirkungen der Standortanalyse.

### Beratungsvorgehen

Das Beratungsvorgehen gliederte sich in mehrere Schritte: Ziele und Erwartungen wurden anfangs geklärt, mithilfe der Fragekarten wurde entlang der Themenfelder reflektiert, Ergebnisse der jeweiligen Termine wurden zusammengefasst, Anregungen des Arbeitsheftes aufgegriffen, eine Einschätzung vorgenommen, das weitere Vorgehen geplant und beim letzten Termin wurde der Beratungsprozess abgeschlossen.

Zu Beginn der Beratungen – wenn notwendig auch im Prozess – erwies es sich als wichtig, den Zweck der Standortanalyse zu verdeutlichen. War der Zweck unklar, verleitete dies die Teilnehmenden dazu, bereits während der Beratung in die operative Arbeit einzusteigen und die Beratungen damit inhaltlich zu überfrachten. Das zugehörige Arbeitsheft von Beginn an einzuführen, stellte sich hierfür und auch insgesamt als hilfreich heraus. Die Teilnehmenden bewerteten es als übersichtliche Informationsquelle. Zudem erwies es sich als hilfreich, dass beim ersten Beratungstermin der zeitliche Umfang des Beratungsprozesses in der Gruppe thematisiert und gemeinsam die Abfolge der Themenfelder festgelegt wird, verbunden mit der konkreten Festlegung der folgenden Termine. Die Erfahrungen der durchgeführten Termine zeigten, dass durchschnittlich 3 Termine zu je 3 h notwendig waren, um die Themenfelder zu besprechen. Pro Termin wurden jeweils 2 Themenfelder bearbeitet.

Die Evaluationsergebnisse zeigten auch, dass es sich empfiehlt, die Standortanalyse flexibel zu handhaben – je nach Stand der Teilnehmenden und Situation im Sozialraum. Wurde in den Beratungen bspw. deutlich, dass Themenfelder bereits an anderer Stelle ausreichend diskutiert wurden, erwies es sich als sinnvoll, von der vorgegebenen Struktur abzuweichen. Eine*r der Teilnehmenden drückte es so aus: „was ist eben in der Gruppe dran und was ist an dem Ort dran und nicht so sehr sich an dem Instrument zwanghaft festzuhalten“. Dennoch wurde die strukturierende Funktion der Arbeit mit den Themenfeldern und Fragekarten von den Beteiligten auch geschätzt, denn statt „abzuschweifen“ hat es „auch immer wieder zurückgeführt zu der Frage“. Resümierend stellte eine*r der Teilnehmenden fest: „das war einfach nochmal gut sich so zu fokussieren“. Hier gilt es, das zeigten unsere Ergebnisse, eine Balance zu finden zwischen dem Zulassen eines freien Gesprächsflusses und dem Vorgehen entlang der vorgegebenen Struktur. Die Teilnehmenden meldeten uns zurück, dass sich die Themenfelder teilweise überschnitten. Es zeigte sich in den Beratungen aber auch, dass Überschneidungen an manchen Stellen durchaus sinnvoll sind, da eine Trennung in der Praxis zumeist nicht existiert und (scheinbare) Wiederholungen weitere wichtige Aspekte in die Diskussion brachten.

Um die einzelnen Beratungstermine abzuschließen, hat es sich in der Praxis bewährt, wesentliche Aspekte zusammenzufassen und gemeinsam mit den Teilnehmenden festzuhalten, welche nächsten Schritte in der Standortanalyse – oder im letzten Termin: beim Auf- und Ausbau einer IKS – anstehen. Die Anregungen für jedes Themenfeld, die die Standortanalyse bereithält, wurden hierbei als unterstützend empfunden. Die sichtbare Dokumentation des bereits Erarbeiteten, der vereinbarten Aufgaben und der Zuständigkeiten in einem Protokoll durch die Berater*in war v. a. am Ende des Prozesses von hoher Relevanz. Das Protokoll bietet, wie ein*e Teilnehmende fand, „einfach eine super Orientierung“ und hilft alles „zusammen[zu]führen, so diese vielen Enden zu einem Ende, dass man sowas wie eine Strategie entwickeln kann“*.*

Zum Abschluss des gesamten Beratungsprozesses erwies es sich in der Praxis zudem als hilfreich, einen Ausblick auf den Gesamtprozess zum Auf- und Ausbau von IKS zu geben sowie mögliche weiterführende Angebote zur Prozessbegleitung zu benennen. Es erwies sich als günstig, verbindliche Vereinbarungen zum Einstieg in die nächste Phase einer IKS zu treffen. Des Weiteren sollten die Berater*innen im letzten Termin überprüfen, ob die Ausgangserwartungen der Teilnehmenden erfüllt wurden, Fragen offengeblieben sind, und sich ein Feedback einholen.

### Beratungskontext

Die Evaluation bestätigte, dass es sich bewährt, die Beratungen mit der Standortanalyse in ein bestehendes Qualifizierungs- und Fortbildungskonzept einzubetten. In Hamburg wurde das Beratungsangebot in das Qualifizierungskonzept der KGC integriert. Die Teilnehmenden bestätigten den Zeitpunkt der Beratungen mit der Standortanalyse als „sehr gut gewählt“. Ihnen erschien die Durchführung der Standortanalyse vor der konkreten Planung einer IKS sinnvoll, „weil man natürlich gerade am Anfang auch noch sehr viele Pflöcke einschlagen will“. Die Standortanalyse hat in ihrer Wahrnehmung „insgesamt so einen ganz guten Startschuss gegeben“. Wichtig wäre aus Sicht der Teilnehmenden, dass klar ist, wer sich der in den Beratungen ausgehandelten nächsten Schritte annimmt und damit den Einstieg in die Initiierungsphase einer IKS gewährleistet.

Für den Beginn der Beratungen mit der Standortanalyse ist eine koordinierende Fachkraft als Bindeglied zum jeweiligen Sozialraum von zentraler Bedeutung. An ein Erstgespräch mit der lokalen Koordination im Vorfeld der Beratungen schlossen sich Folgetermine in der Gruppe für die eigentliche Standortanalyse an. In der Beratungspraxis erwiesen sich 2 Termine à 3 h mit geringen zeitlichen Abständen zwischen den beiden Terminen als sinnvolle Balance zwischen den knappen zeitlichen Ressourcen der Akteur*innen und dem Diskussionsbedarf zur gemeinsamen Einschätzung des Sozialraums. Eine gleichbleibende Gruppenzusammensetzung mit bis zu 4 zentralen Akteur*innen des Sozialraums bewährte sich, auch wenn es aus Sicht der Teilnehmenden „natürlich gut [wäre], wenn alle, die damit zu tun haben, dabei wären“ – aber dann wäre „das Instrument auch überfordert“. Dabei können i. d. R. nicht alle Bereiche des Sozialraums abgedeckt werden. Es empfiehlt sich daher, mit einer Art Steuerungsgruppe zu arbeiten, die gut im Sozialraum vernetzt ist.

Deutlich wurde die zentrale Rolle der externen Beratung während des Standortanalyseprozesses. Nach unseren Ergebnissen sollte ein*e Berater*in, die/der nicht Teil der Struktur vor Ort ist und daher unabhängig und neutral, diese Rolle übernehmen. Die Teilnehmenden begründeten diese Einschätzung damit, dass eine interne Moderation solcher Prozesse „auch öfter schon mal versucht [wurde] und das hatte dann trotzdem andere Ergebnisse“. Aus der Sicht der Teilnehmenden wäre es „wirklich noch was anderes, wenn das halt wirklich so von außen kommt“. Ein*e externe*r Berater*in unterstützt aus Sicht der Teilnehmenden das gemeinsame Reflektieren, das „wie eine Supervision“ ist. Die Ergebnisse zeigten deutlich, welche Aufgaben Berater*innen in den Beratungsprozessen mit der Standortanalyse übernehmen: Sie moderieren und strukturieren den Austausch, halten zugleich dessen Ergebnisse fest, geben bei Bedarf Informationen und/oder setzen Impulse. Sie bereiten zudem die Beratungstermine vor und nach.

### Wirkungen der Standortanalyse

Die Evaluation zeigte, neben den dargestellten Erkenntnissen zum Beratungskontext und -vorgehen, auch die Wirkungen der Arbeit mit der Standortanalyse. So fanden wir heraus, dass das Instrument dem Austausch der Akteur*innen vor dem Start einer IKS den notwendigen Raum und die passende Struktur bietet. Aussagen der Teilnehmenden zufolge berücksichtigt die Standortanalyse alle wichtigen Themen. Die Teilnehmenden fühlten sich in dem Prozess „geleitet“ und schätzten, dass ihnen durch die Beratungen ein kommunikativer Raum geöffnet wurde, „sich wirklich intensiv“ mit dem Aufbau einer IKS auseinanderzusetzen. Insbesondere die externe Moderation wurde von den Teilnehmenden als bedeutend hervorgehoben, denn sie ermöglichte einen Austausch, „ohne dass man selber darauf achten muss, das zu strukturieren oder zu moderieren oder den Faden zu behalten“.

Die Beratungen mit der Standortanalyse ermöglichten, vor dem Start einer IKS das notwendige Wissen zusammenzutragen und alle auf denselben Stand zu bringen, zudem Wissenslücken zu identifizieren. Im Ergebnis, das meldeten die Teilnehmenden zurück, entstand so ein für den Fortgang des Prozesses wichtiger Gesamtüberblick über relevante Gegebenheiten des Sozialraums, bspw. vorhandene Angebote, Strukturen, Akteur*innen oder/und Ressourcen. Der Beratungsprozess mit der Standortanalyse konnte, das spricht aus den Interviews und Gruppendiskussionen, bei den einzelnen beteiligten Akteur*innen das Verantwortungsgefühl für die Gesundheitsförderung im Sozialraum stärken. Durch das gemeinsame Herausarbeiten der nächsten relevanten Schritte und der hierfür notwendigen Ressourcen entstanden konkrete Aufträge und verbindliche Zusagen. Wie die befragte Beraterin erklärte, wurden „einzelne Punkte mitgenommen … und daran weitergearbeitet“. Eine wichtige Wirkung ist die Stärkung der Vernetzung der Akteur*innen untereinander. Sie kann, wie auch die zuvor benannten, wiederum künftige Wirkungen erzeugen.

## Diskussion

Dieser Beitrag verfolgte die Frage, wie der Transfer partizipativer Forschungsergebnisse in die Praxis gelingen kann. Im Rahmen von KEG haben wir mit der Standortanalyse eben diesen Praxistransfer forciert. Insgesamt ist das Ziel von PGF, neben neuen Erkenntnissen, die Veränderung der Praxis hin zu mehr gesundheitlicher Chancengleichheit [13]. An diesem doppelten Anspruch partizipativer Forschungsvorhaben [30] setzten wir in der Arbeit mit der Standortanalyse an.

Als Wirkungen auf die Praxis kommunaler Gesundheitsförderung, die auf der Anwendung der Standortanalyse als gemeinsam generiertes Ergebnis eines partizipativen Forschungsprozesses basieren – im Diskurs von PGF als kollaborative Wirkungen bezeichnet [31] –, sind v. a. die Stärkung des Austausches von Fachkräften und die damit einhergehende Vernetzung, die Schaffung gegenseitiger Transparenz sowie das Strukturieren und Handhabbar(er)machen der Entwicklung einer IKS zu nennen. Die zentrale Herausforderung im Auf- und Ausbau von IKS ist es, Fachkräfte verschiedener Ressorts in einen Diskussions- und Abstimmungsprozess zu bringen. Dieser Herausforderung wird durch die Standortanalyse begegnet. Das Instrument leistet damit einen Beitrag dazu, die Voraussetzungen für Bedingungen zu schaffen, die das Wohlbefinden und die Lebensqualität von Menschen in ihrem Wohnumfeld durch integriertes Handeln fördern. Der zentrale Wirkungsweg hierbei ist die Partizipation derjenigen, die an und mit IKS arbeiten. Wie dargestellt, waren bereits die Grundlagen der Entwicklung der Standortanalyse ein Ergebnis und eine Erkenntnis eines partizipativ gestalteten Forschungsprozesses.

Auf Basis der Rückmeldungen aus der Praxis im Rahmen der Evaluation wurde die Standortanalyse weiterentwickelt. So konnte sie (noch) stärker an die Bedarfe der Praxis angepasst und der bundesweiten Gemeinschaft von Praktiker*innen der Gesundheitsförderung zugänglich gemacht werden. Es wurde bspw. das vorgeschlagene Beratungsvorgehen angepasst, das Arbeitsheft und einzelne Fragekarten zur Standortanalyse überarbeitet sowie darin ein Leitfaden für Berater*innen, der sich aus den Ergebnissen der Erprobungsphase in Hamburg speist, ergänzt.

Zudem wurde die zentrale Rolle der Moderation bei der Standortanalyse in den Ergebnissen der Evaluation sehr deutlich. Qualifikationen, die wir daher für die Beratung mit der Standortanalyse für wichtig halten, sind Erfahrungen mit Gesprächsführung und Beratung sowie in der Moderation und Dokumentation von Prozessen. Fach- und Methodenwissen aus den Bereichen Gesundheitsförderung, Organisationsentwicklung und Prozessbegleitung sind ebenso bedeutsam wie die Kenntnis der (kommunalen) Strukturen, der Gegebenheiten und Fördermöglichkeiten vor Ort. Es empfiehlt sich darüber hinaus, mit dem Aufbau und den Inhalten der Standortanalyse vertraut zu sein.

## Fazit

Bereits das Beratungsinstrument der Standortanalyse entstand in einem partizipativen Forschungsprozess zwischen Hochschule und Praxispartner*innen. Bei seiner Entwicklung wurden also die Expertise und Erfahrung der Praxis hinzugezogen sowie deren Bedarfe berücksichtigt – ein Gelingensfaktor für den Transfer. Die Evaluation der Erprobungsphase führte zu wichtigen, praxisrelevanten Veränderungen und stärkte so wiederum den Transfer der Forschungsergebnisse in die Praxis. Die Evaluationsergebnisse rücken insgesamt die Bedeutung der Prozessbegleitung beim Auf- und Ausbau von IKS ins Licht. Akteur*innen benötigen hierfür einen Rahmen und Hilfestellung. Dies weist auf weitere Entwicklungsbedarfe von handhabbaren Instrumenten für die Praxis kommunaler Gesundheitsförderung hin. Von zentraler Bedeutung für die Arbeit mit solchen Instrumenten ist, das wurde im Beitrag deutlich, eine externe Beratung und Begleitung. Es gilt zu überlegen, ob und wie eine solche Begleitung noch stärker in Strukturen, wie bspw. die KGC, eingebettet werden kann, welche Qualifizierung hierfür benötigt wird bzw. wie die Nutzung in Form eines Multiplikator*innen-Konzeptes aussehen könnte. Die HAG konzipiert zu diesem Zweck ein Schulungsangebot. Digitale Formate werden wie bereits erwähnt derzeit entwickelt.
